# Dextran: Influence of Molecular Weight in Antioxidant Properties and Immunomodulatory Potential

**DOI:** 10.3390/ijms17081340

**Published:** 2016-08-19

**Authors:** Vinicius C. Soeiro, Karoline R. T. Melo, Monique G. C. F. Alves, Mayara J. C. Medeiros, Maria L. P. M. Grilo, Jailma Almeida-Lima, Daniel L. Pontes, Leandro S. Costa, Hugo A. O. Rocha

**Affiliations:** 1 Departamento de Bioquímica, Universidade Federal do Rio Grande do Norte (UFRN), Av. Salgado Filho 3000, Natal-RN 59078-970, Brazil; vihcampelo@gmail.com (V.C.S.); melo.krt@gmail.com (K.R.T.M.); monique.gabi@gmail.com (M.G.C.F.A.); mlporpino@hotmail.com (M.L.P.M.G.); biolottus23@yahoo.com.br (J.A.-L.); leandro-silva-costa@hotmail.com (L.S.C.); 2 Instituto de Química (IQ), Universidade Federal do Rio Grande do Norte (UFRN), Av. Salgado Filho 3000, Natal-RN 59078-970, Brazil; mayarajane20049@hotmail.com (M.J.C.M.); pontesdl@yahoo.com (D.L.P.); 3 Instituto Federal de Educação, Ciência a Tecnologia do Rio Grande do Norte (IFRN), Av. Planalto, Km 406—Planalto, Ceará-Mirim-RN 59580-000, Brazil

**Keywords:** α-d-glucans, *Leuconostoc mesenteroides*, immunomodulatory activity, 40 kDa dextran, antioxidant activity

## Abstract

Dextrans (α-d-glucans) extracted from *Leuconostoc mesenteroides*, with molecular weights (*M*_W_) of 10 (D10), 40 (D40) and 147 (D147) kDa, were evaluated as antioxidant, anticoagulant and immunomodulatory drugs for the first time. None presented anticoagulant activity. As for the antioxidant and immunomodulatory tests, a specific test showed an increase in the dextran activity that was proportional to the increase in molecular weight. In a different assay, however, activity decreased or showed no correlation to the *M*_W_. As an example, the reducing power assay showed that D147 was twice as potent as other dextrans. On the other hand, all three samples showed similar activity (50%) when it came to scavenging the OH radical, whereas only the D10 sample showed sharp activity (50%) when it came to scavenging the superoxide ion. D40 was the single dextran that presented with immunomodulatory features since it stimulated the proliferation (~50%) of murine macrophages (RAW 264.7) and decreased the release of nitric oxide (~40%) by the cells, both in the absence and presence of lipopolysaccharides (LPS). In addition, D40 showed a greater scavenging activity (50%) for the hydrogen peroxide, which caused it to also be the more potent dextran when it came to inhibiting lipid peroxidation (70%). These points toward dextrans with a 40 kDa weight as being ideal for antioxidant and immunomodulatory use. However, future studies with the D40 and other similarly 40 kDa dextrans are underway to confirm this hypothesis.

## 1. Introduction

Glucans are polysaccharides of d-glucose monomers linked by glycosidic bonds. Despite the concept, the definition of a glucan is not yet fully established since some authors still refer to heteropolysaccharides that are high in glucose content as glucans [1]. In spite of having a monotone monosaccharide composition in comparison to other polysaccharides, glucans show variation when it comes to their molecular weights, types of glycosidic links, anomeric configurations of the glucose residue and types and degrees of ramifications throughout their chain [2].

These biopolymers are synthesized mainly by fungi, bacteria, plants and algae [3]. In these organisms, glucans perform a widely structural role and are, in an array of cases, the most widely available component in the cellular walls [4]. It is worth highlighting that, in some cases, bacteria and fungi may use glucans as an energy source [5]. 

There are α-, β- and αβ-glucans. The most widely available are, however, α- and β-glucans [6]. β-glucans are described as presenting with structural variations in numeric correlation to the links between their monomers. Those can be type β-(1→3), β-(1→4) or β-(1→6), with possible ramifications throughout the chain [7]. These polymers have been undergoing investigations as to their biological, physiological and pharmacological potentials both in vitro and in vivo. Data already point toward their possible use in treating different types of cancer due to their antitumor [8], anti-proliferative [9] and immune response modulating [10,11] potential. In addition, the pharmacological potential of β-glucans may also be availed in other areas since they can behave as anti-nociceptive [12] and antioxidant [13] agents.

The α-glucans whose monosaccharides present exclusively in the α configuration are widely used as an energy source for living organisms. As an example, one can cite the α-glucans known as glycogen (a source of energy for animals) [14] and starch (α-glucan used as a source of energy by plants) [15]. However, α-glucans can also present with other interesting properties such as anti-inflammatory, antimicrobial [16], immunomodulatory [17] and antioxidant [18]. Not all α-glucans, however, have been extensively evaluated for their pharmacological potential. One example of that are the dextrans. 

Several groups showed the relationship between molecular weight (*M*_W_) and pharmacological activities of polysaccharides including fucans [19], alginates [20], hyaluronic acid [21] and glucans. For instance, Zho et al. [22], working with two β-glucans, showed that high molecular weight β-glucan (552 kDa) inhibits differentiation of 3T3-L1 pre-adipocytes stronger than that of low molecular weight β-glucan (32 kDa). Dextran sulfates (DS) are also affected by their molecular weight, and DS with three different *M*_W_ (4, 15 and 40 kDa) were used as anti-aggregation agent on cell growth and monoclonal antibody (mAb) production and the data showed that the 40 kDa DS was the most effective in attenuating cell aggregation and showed the highest maximum mAb concentration [23]. However, there are no data regarding dextrans.

Dextrans are described as α-glucans with type α-(1→6) links in their main chains and type α-(1→3) (the most common), α-(1→4) or α-(1→2) ramifications [24]. They also present with a variety of molecular weights going from 1 to 2000 kDa [25]. These polymers are synthesized mainly by fungi and bacteria of the *Leuconostoc* genus [26]. However, very little is known of the biological activities of dextrans, and those that are known are described solely when the dextrans are associated with other molecules [27]. Dextrans with different molecular weights synthesized by *Leuconostoc mesenteroides* (*L. mesenteroides*) are produced in large quantities and obtained in a commercial fashion. However, to the best of our knowledge, there has not yet been a study to evaluate the pharmacological activities of these polymers. In this context, three dextrans with different *M*_W_ (10, 40, and 147 kDa) synthesized by the *L. mesenteroides* were obtained commercially and evaluated as to their antioxidant, immunomodulatory and anticoagulant activities.

## 2. Results and Discussion

### 2.1. Glucan Characterization

The glucans called D10, D40 and D147 were acquired commercially and the information made available by the company selling said glucans lead one to believe they are the same compound, being differentiated by the molecular weight each one presents. In this particular stance, D10 presents with 10 kDa, while D40 and D147 present with 40 and 147 kDa, respectively. We chose these dextrans because they represent dextran molecules with low, medium and high molecular mass, respectively, in agreement with other papers that evaluated the influence of the molecular weight in the polysaccharides activity [19,20,21,22,23]. In addition, we did not used dextrans of higher molecular weights like 300 or 500 kDa or even higher because they showed very low water solubility, as well as because they could break during the tests and their fragments could alter the overall result. With the intent of confirming the identities of said glucans, as well as of verifying if any contamination has occurred by impurities that might mask results, we conducted a series of chemical analyses of infrared spectroscopy (FTIR).

### 2.2. Glucan Fourier Transform Infrared Spectroscopy (FTIR) Analyses

**Figure 1:**
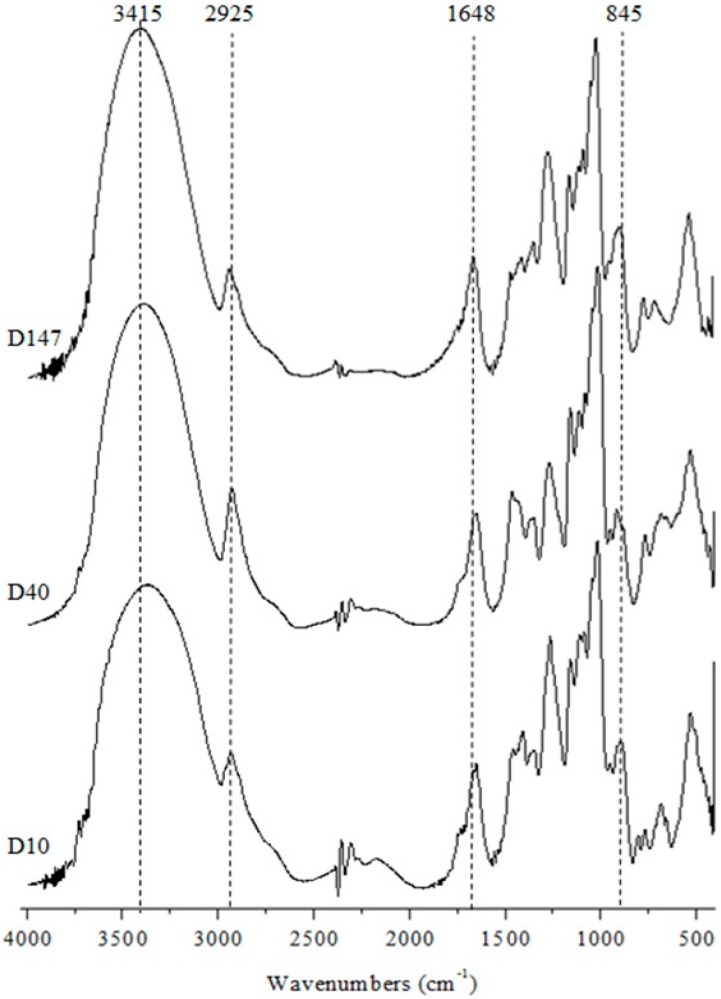
Fourier transform infrared spectroscopy (FTIR) spectra of the dextrans. The characteristics signals are in evidence for the regions between 4000 and 400 cm^−1^.

Fourier Transform Infrared Spectroscopy (FTIR) Analyses is a technique that can be executed rapidly and allows for trustworthy confirmation of the molecule identity. Therefore, to confirm that samples in fact are dextrans and pure the samples were submitted to FTIR analysis and the spectra obtained can be seen on Figure 1. As can be observed, regardless of molecular weight, glucans D10, D40 and D147 present with very similar spectra, which indicates they are the same compound. Another important fact is that the spectra obtained from the three samples are the same obtained from other glucans [28].

With regards to the main signals noted it can be observed that one strong band in the 3415 cm^−1^ region corresponds to the asymmetric stretching O–H that overlaps itself over the hydrogen intramolecular link signals [29]. One signal between 2925 and 2932 cm^−1^ can be attributed to C–H symmetric and asymmetric stretching, respectively [17]. There is a signal in the region around 1648 cm^−1^ that corresponds to the water solvation layer around the polysaccharide [30]. These signals are characteristic to a number of polysaccharides such as chitosans [31], galactans [32], and glucans [33]. Other characteristic signals of glucans were identified, such as those on the 1457 and 1277 cm^−1^ regions that correspond to the signals of the glycosidic units, signal around 1156 cm^−1^ that corresponds to the C–O–C asymmetric stretching; signal around cm^−1^ that corresponds to C–C [34]; and signals around 915 and 845 cm^−1^ that indicate the presence of α-glycosidic links [35]. These signals were also identified in other dextran spectra [36] and, therefore, confirm the D10, D40 and D147 samples are dextrans. It is notable that no signs of protein content were found. 

### 2.3. Chemical Analyses

All three samples, D10, D40 and D147, were analyzed as to the presence of contaminants: proteins and phenolic compounds. The data are available in Table 1. It can be observed that the presence of these was not identified in the samples. The information is important since both proteins and phenolic compounds are molecules that can influence in biological systems [37,38], which could create doubts as to possible activities that might come to be observed for the D10, D40, and D147 glucans.

As it relates to the total amounts of sugars, it was verified that D10, D40 and D147 possess an elevated amount of this compound (~90%). It should be pointed out that the added percentage of total amounts of sugars, proteins and phenolic compounds does not account to 100 percent. This fact was also noted in the chemical composition analyses of other sugars such as ramnanas and fucans [39], and can be explained as decurrent from the hygroscopic property of polysaccharides since, even after lyophilization, they are capable of absorbing moisture from the environment extremely rapidly [40].

Dextrans used in the study are composed exclusively by glucose and, therefore, are homoglucans. *Leuconostoc mesenteroides* can synthesize, beyond dextrans, a small amount of heteroglucans that contain residue of mannose and galactose [34]. However, as we have not identified other monosaccharides besides glucose, we conclude that D10, D40 and D147 are dextrans with a high degree of purity.

### 2.4. Antioxidant Activities

Antioxidants are described mainly as low molecular weight molecules that have a protective effect both against non-reactive species, such as the hypochlorite, and against reactive oxygen species (ROS) and reactive nitrogen species (RNS) [41].

The formation process of these two reactive species is done through a chain reaction involving three steps (initiation, propagation and termination) in which the antioxidants take effect through a series of mechanisms. Thus, different methods were used to evaluate the effect of dextrans D10, D40 and D147 at the different stages: initiation (total antioxidant capacity and reducing power), propagation (chelation of copper and iron) and termination (scavenging of the hydroxyl superoxide radical and of the hydrogen peroxide). Moreover, the inhibiting lipid peroxidation of the dextrans was also determined.

#### 2.4.1. Chelating of Copper and Iron Ions Assay

**Figure 2:**
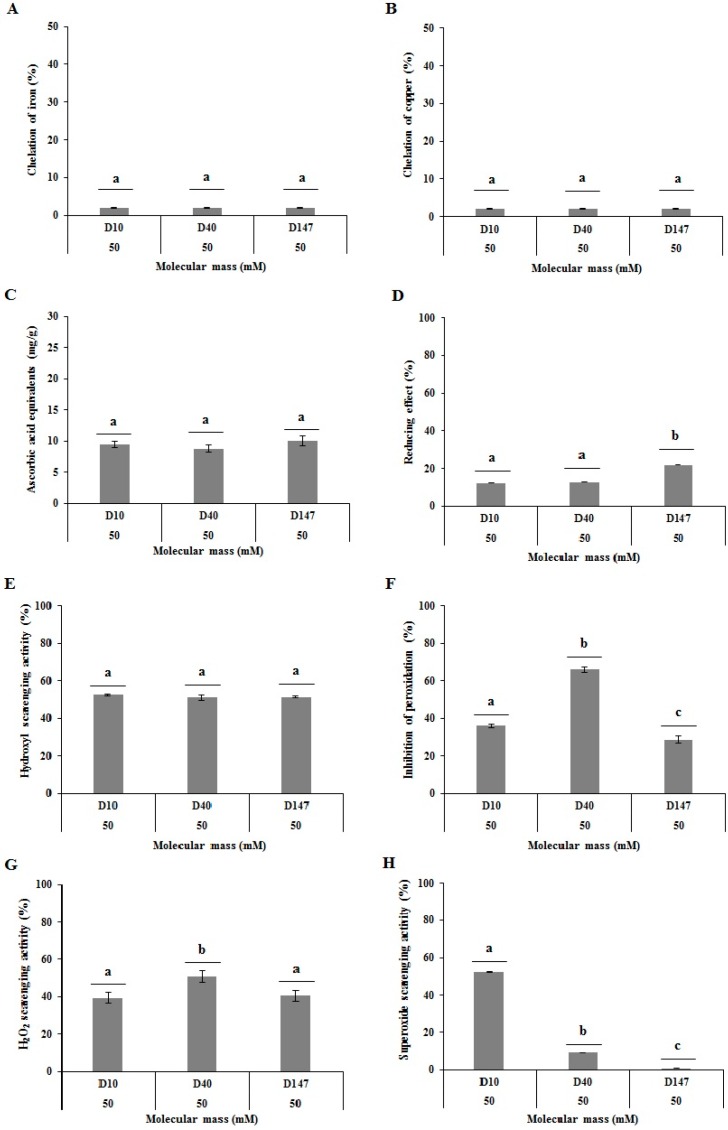
Antioxidant activities of D10, D40 and D147. (**A**) Ferrous chelating; (**B**) copper chelating; (**C**) total antioxidant capacity; (**D**) reducing Power; (**E**) hydroxyl radical scavenging; (**F**) inhibiting lipid peroxidation; (**G**) hydrogen peroxide radical scavenging; and (**H**) superoxide radical scavenging. Letters ^a,b,c^ represent the significant difference between the samples by the simple variance analyses (one-way ANOVA) followed by the Tukey–Kramer (*p* < 0.05) test.

The D10, D40 and D147 dextrans presented with no chelating activity of Fe^2+^ and Cu^2+^ ions (Figure 2A,B). It was not possible to compare the results to those presented by other authors since no article was identified evaluating this specific activity in dextrans. With regards to other glucans, despite few records, it was verified that the activity occurs and that it is detached from the molecular weight, since α-glucans of 9 and 113 kDa extracted from *Aconitum kusnezoffii* Reichb tubers presented with a similar chelating activity for iron ions. However, it is noteworthy that the activity was only 10% [18]. Low chelating activity of iron was also identified in 5 and 15 kDa dextrans extracted from the *Ganoderma lucidum* mushroom fruiting body. In this case, authors pointed out a chelating activity of 50%. This activity was only achieved, however, when glucans were at a concentration of 10.0 mg/mL [42], meaning a much superior concentration in comparison to what was used in this paper. For comparison purposes, corncob xylans presented with a chelating iron activity around 80% in a 1.0 mg/mL concentration [43]. To summarize, data seen here lead to the proposition that metal chelating activity seems not to be the main antioxidant mechanism in glucans.

#### 2.4.2. Reducing Power and Total Antioxidant Capacity (TAC) Assays

Tested dextrans presented a total antioxidant capacity (TAC) around 9.8, 8.7 and 9.9 equivalent in ascorbic acid milligrams to D10, D40 and D147, respectively, which is the first report of glucans with a TAC activity (Figure 2C). In reducing power test, the data were expressed as percentage activity of ascorbic acid control at 0.1 mg/mL. For the reducing power, dextrans D10 and D40 presented with 12.1% and 12.8% activity, respectively, while D147 reached a superior activity around 21% (Figure 2D). These reducing power values were similar to those obtained in other glucans, particularly β-glucans such as laminarin (extracted from the *Laminaria digitate* algae), botryosphaeran (extracted from the *Botryosphaeria rhodina* MAMB-05 fungi), curdlan (extracted from the *Alcaligenes faecalis* bacteria) and lasiodiplodan (extracted from the *Lasiodiplodia theobromae* MMPI fungi) [44].

In these two tests, one evaluates the samples’ capacities to donate electrons in a solution. For the polysaccharides, the capacity was possibly connected to the presence of hydroxyls linked along the entire chain [39]. However, it can be observed that this capacity in dextrans, as in other molecules, is dependent on the experimental conditions since the TAC dextran tests present with a much smaller activity than the one observed in the reducing power test. The fact that the dextrans presented with reducing activity in two tests that have different chemical environments indicates these molecules can also present the activity in the different chemical microenvironments found inside the cells.

Specifically, in regards to the reducing power test, it was verified that the D147 dextran presented with double the activity of the other dextrans (D10 and D40), which indicates the reducing capacity of the dextrans is dependent on molecular weight. However, it is notable that no other studies that evaluated the correlation between molecular weight in glucans and reducing power were found. Therefore, it would be necessary for other studies with glucans of varied molecular weights be conducted to corroborate data found.

#### 2.4.3. Hydroxyl Radical Scavenging Assay

Dextrans D10, D40 and D147 showed scavenging activity for hydroxyl ions of ~50% (Figure 2E). The activity was higher than the observed in a β-glucan obtained from the *Dictyophora indusiata* fungi, which was ~39% of scavenging [45]. However, as it relates to molecular weight, it was possible to observe the activity in dextrans is not influenced by this property. This, in turn, corroborates data described by Hong and collaborators, which show β-glucans with very distinctive weights, such as 70 and 900 kDa obtained from the *Paenibacillus polymyxa* JB115 bacteria, present with the same scavenging capacity for hydroxyl radicals [8]. 

#### 2.4.4. Inhibiting Lipid Peroxidation Assay

When testing for the dextrans capacity to inhibit the lipid peroxidation it was observed that D10 and D147 presented with inhibiting capacity of ~30%, while D40 presented with an elevated capacity of ~70%. The amount obtained with D40 becomes significant when compared to those found in the literature (Figure 2F). For example, homoglucans extracted from the *Ganoderma lucidum* fungi inhibited the peroxidation by around 77%, while heteroglucans extracted from the *Agaricus bisporus* fungi inhibited lipid peroxidation by merely 46%. In both cases, however, values were only obtained at 20 mg/mL [46], which is much greater than the concentration used in our experiments. These authors also state that the difference between the two glucans occurred due to the difference in the monosaccharide composition, meaning the presence of other monosaccharides other than glucose would lead heteroglucans to being less effective. The data lead to the belief that homoglucans have a good potential to prevent lipid peroxidation. 

Data also lead to the observation that dextrans are more active in certain molecular weights than in others, as if there were an ideal weight for a dextran that would lead it to show their maximum lipid peroxidation inhibition capacity, since D40 was much more effective than the other dextrans tested. In addition, one other dextran used in the clinic called Dextran 40 has already presented itself as a lipid peroxidation inhibition agent, and the molecular weight of this dextran is also approximately 40 kDa [47]. New studies that compare other glucans with a molecular weight around 40 kDa to glucans with larger or smaller weights will be able to confirm this hypothesis.

This lipid peroxidation inhibition capacity observed in the dextrans stands out since there is a clear correlation between aging, caused by the continuous and elevated exposure to reactive species, and lipid peroxidation. This correlation was made clear once the accumulation of lipofuscin, a pigment capable of detecting the presence of free radicals and lipid peroxidation, was observed in the development of atherosclerosis [48]. In addition, the increased formation of lipid peroxides is also observed in Alzheimer’s disease, cancer, rheumatoid arthritis, and other immune diseases [49]. Therefore, inhibiting the lipid peroxidation could promote a certain protection to the body, once it is clearly related to the chain reaction that causes selective changes to the cell’s signals, protein damage and DNA.

#### 2.4.5. Hydrogen Peroxide Radical Scavenging Assay

With the intent of better understanding the dextran’s capacity to inhibit the lipid peroxidation, an assay was conducted to measure the scavenging of hydrogen peroxide radicals, since this particular radical is closely related to reactions that form the peroxide radicals [44]. Dextrans D10 and D147 presented with a scavenging capacity of ~40%, while D40 showed a scavenging capacity of 50% (Figure 2G). This result was bigger than that obtained with 140 kDa glucans extracted from the roots of *Aconitum kusnezoffii* Reichb, which presented with a 33% capacity [18], as well as two glucans of 5 and 15 kDa obtained from the *Ganoderma lucidum* fungi that showed a scavenging capacity of around 30% [42]. However, we highlight the fact that, just as for the lipid peroxidation assay, the D40 dextran was more effective than the D10 and D147, which shows the main inhibition mechanism for lipid peroxidation in dextrans is in their capacity to scavenge the hydrogen peroxide.

#### 2.4.6. Superoxide Radical Scavenging Assay 

With regards to the dextran’s capacity to scavenge the superoxide ions, it was observed that the activity decreases as the molecular weight of the dextran increases, since D147 presented with a 0.7% activity, D40 with 9% activity, and D10 presented with a 52.3% activity (Figure 2H). The data corroborate Liu and collaborators observations that showed a 5.2 kDa glucan from the *G. lucidum* fungi showed a scavenging activity of around 80%, while only ~50% was obtained from a 15 kDa from the same fungi [42].

In short, the data lead to the proposition that dextrans D10 and D40 are more promising molecules from an antioxidant point of view, when compared to the D147. In addition, still favoring D10 and D40, the search is always for lower molecular weight compounds since those are more easily absorbed and distributed inside the human system, metabolized and discarded in urine [50].

### 2.5. 3-(4,5-Dimethylthiazol-2-yl)-2,5-diphenyltetrazolium bromide (MTT) Mitochondrial Reduction Assay

**Figure 3:**
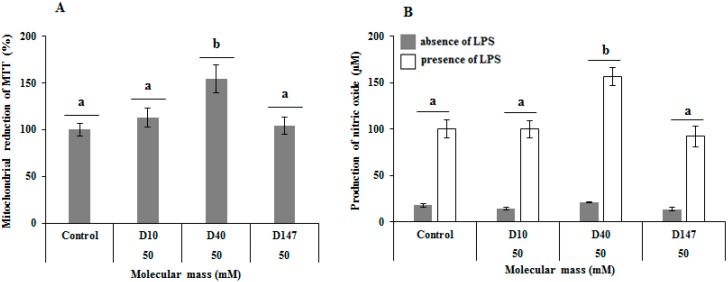
Effect of D10, D40 and D147 in RAW cells: (**A**) 3-(4,5-dimethylthiazol-2-yl)-2,5-diphenyltetrazolium (MTT) mitochondrial reduction by cells in the presence of glucans; and (**B**) nitric oxide production by RAW cells in the presence of glucans. Letters ^a,b^ represent the significant difference between the various samples according to the simple variation analyses (one-way ANOVA) followed by the Tukey–Kramer (*p* < 0.05) test. LPS, lipopolysaccharides.

Dextrans D10, D40 and D147 allowed for an increase in the 3-(4,5-dimethylthiazol-2-yl)-2,5-diphenyltetrazolium bromide (MTT) reducing activity in RAW 264.7 cells (murine macrophages) (Figure 3A). However, the effect was only significant in the presence of D40, which points to a mitosis action in the dextran. The data suggest that the dextrans in this study do not show cytotoxic activity to the cells. Other glucans are described as having a mitosis action in macrophages [5] and lymphocytes [18,51].

### 2.6. The Effect of Dextrans in the Nitric Oxide Production in RAW Cells

β-Glucans are cited as being immunomodulatory agents in a variety of studies [44,51,52]. However, there are few data regarding α-glucans and we could not find any data referring to the evaluation of the immunomodulatory ability of dextrans. Since D10, D40 and D147 dextrans were not cytotoxic to RAW cells, it was evaluated if they would affect the production of nitric oxide by those cells, both in the presence and absence of LPS (a widely known macrophage activator). Data showed, however, that D10 and D147 dextrans did not affect the nitric oxide (NO) production by the RAW cells in every tested condition. On the other hand, D40 decreased ~40% the amount of NO released by RAW cells, both in the presence and absence of LPS (Figure 3B).

In short, data obtained here lead to the observation that D10 and D147 dextrans are not immunomodulatory agents. However, D40 stimulates the proliferation of macrophages and inhibits the production of NO, which awards this dextran an immunomodulatory action. This also leads to the observation that, as well as for the antioxidant action, the dextrans with molecular weight around 40 kDa will tend toward more noticeable action.

### 2.7. Anticoagulant Activities (Activated Partial Thromboplastin Time (aPTT) and Prothrombin Time (PT))

**Figure 4:**
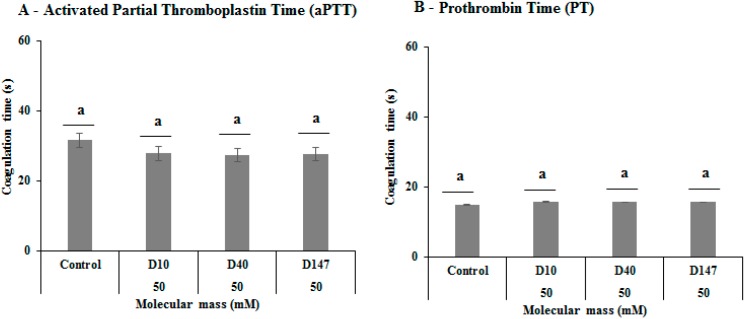
Anticoagulant activities of D1, D40 and D147: (**A**) activated partial thromboplastin time (aPTT); and (**B**) prothrombin time (PT). Letter ^a^ represent non-significant difference between the various samples according to the variation analyses (one-way ANOVA) followed by the Tukey–Kramer (*p* < 0.05) test.

Dextrans D10, D40 and D147 did not show anticoagulant activity, neither in activated partial thromboplastin time (aPTT) nor prothrombin time (PT) assays (Figure 4A,B). These results corroborate with those observed in the PT and aPTT assays performed with 420 kDa β-glucan from lichen *Parmotrema mantiqueirense* Hale, which was also not able to induce any apparent coagulation. On the other hand, this glucan showed anticoagulant activity when it was sulfated [53]. The data found with dextran D10, D40 and D147 are positive because anticoagulant activity could be an obstacle to the use of these dextrans as antioxidant or immunomodulatory agents.

## 3. Materials and Methods

### 3.1. Materials

The 10, 40 and 147 kDa dextrans (Catalog number: 1179876, 1179865 and D-487); bovine serum albumin (BSA); sodium chloride; ferrozine; butylated hydroxyanisole (BHA); pyrocatechol violet; and ascorbic acid were purchased from Sigma Chemical Co. (St. Louis, MO, USA). Potassium ferricyanide, ferrous sulfate (II), ethylenediaminetetraacetic acid (EDTA), Gallic acid, ammonium molybdate, hydrogen peroxide 30%, acetic acid, Folin–Ciocalteu phenol reagent, ethanol and sulfuric acid were obtained from Merck (Darmstadt, Germany). Culture media components Minimum essential Dulbecco’s modified Eagle medium (DMEM), l-Glutamine, sodium pyruvate, sodium bicarbonate, non-essential amino acids, fetal bovine serum and phosphate buffered saline (PBS) were acquired from Invitrogen Corporation (Burlington, ON, Canada). All other reagents and solvents were of analytical degree.

### 3.2. Fourier Transformed Infrared (FT-IR) Spectroscopy Analysis

The infrared spectra of D10, D40 and D147 were obtained using infrared spectroscopy via Fourier transform (IRAffinity-1 spectrometer, Shimadzu Corp., Kyoto, Japan) equipped with the IRsolution 1.20 software. Samples were mixed completely with the dried potassium bromide powder (KBr) and compressed. The sweep frequency range was 4000–400 cm^−1^. Representative spectra of three independent experiments are shown [54].

### 3.3. Chemical Analysis and Monosaccharide Composition

Total amounts of sugars, proteins and phenolic compounds were determined as described previously [55]. Total amounts of sugars were determined by the phenol-H_2_SO_4_ method using d-glucose as a standard. Amounts of proteins were measured by employing the Coomassie Brilliant Blue reagent, using bovine serum albumin (BSA) as a standard. Phenolic compounds were measured by the Folin–Ciocalteau phenol reagent method, using Gallic acid as a standard. The monosaccharide composition was verified by the acid hydrolysis of the polysaccharides using HCl in a variety of concentrations (0.5, 1.0, 2.0, and 4.0 M) for different periods (0.5, 1, 2, and 4 h) at 100 °C, as described by Camara and collaborators [30]. It was verified that HCl (2.0 M) for a period of 2 h was the best condition available to break down the polysaccharides without degrading the free monosaccharides in the sample. After the acid hydrolysis, the monosaccharide composition was determined by the VWR-Hitachi Lachrom Elite^®^ HPLC system (Hitachi Co., Tokyo, Japan) with the refraction index detector. The LichroCART^®^ 250-4 (250 mm × 40 mm) column connected to a Lichrospher^®^ 100 NH_2_ (5 μm) was then attached to the system. The concentration of the sample used was 50 mM and the analysis time was 25 min. The following sugars were used as reference: galactose, fucose, fructose, a rabinose, xylose, glucose and mannose.

### 3.4. Antioxidant Activities

The antioxidant activities were investigated through a series of eight in vitro assays: iron ion chelation, copper ion chelation, total antioxidant capacity (TAC), reducing power, hydroxyl radical scavenging activity, inhibiting lipid peroxidation, hydrogen peroxide scavenging activity and superoxide radical scavenging activity. All eight tests were conducted with the 50 mM concentration for all samples. All assays were conducted three times, always in triplicate. 

#### 3.4.1. Chelating of Iron

The assay was conducted to investigate the sample’s capacity to chelate iron ions as described earlier [30]. Briefly, the reaction containing ferrous chloride (2 mM) and ferrozine (5 mM) were mixed with the samples and incubated for 10 min at 25 °C. The change in color was measured in a microplate reader (BioTek, Winooski, VT, USA) at 562 nm against a blank. EDTA was used as positive control. The ability of the samples in chelating the iron ion was calculated using the following equation: [(Absorbance of blank − Absorbance of the sample)/Absorbance of the blank)] × 100.

#### 3.4.2. Chelating of Copper

The assay investigated the sample’s capacity to chelate copper ions. Pyrocatechol violet, the reagent used in this assay, has the ability to associate with certain cations. In the presence of chelating agents, this combination is not formed, resulting in decreased staining. The test was performed in 96-well microplates with a reaction mixture containing the samples (50 mM), pyrocatechol violet (4 mM), and copper II sulfate pentahydrate (50 mg/mL). All wells were homogenized with the aid of a micropipette and the solution absorbance was measured at 632 nm against a blank [43]. EDTA was used as positive control. The ability of the samples in chelating the copper ion was calculated using the following equation: [(Absorbance of blank − Absorbance of the sample)/Absorbance of the blank)] × 100.

#### 3.4.3. Total Antioxidant Capacity (TAC)

The total antioxidant capacity assay consists of the reduction of the Mo^+6^ to Mo^+5^ ions by the samples and the subsequent formation of the phosphate–molybdate complex in low pH values. The dextrans and the reagent solution (sulfuric acid 0.6 M, sodium phosphate 28 mM and ammonium molybdate 4 mM) were incubated at 95 °C for 90 min. immediately afterwards, the absorbing capacities of each solution were measured at 695 nm against the blank. The TAC was recorded as ascorbic acid milligrams/dextran grams, described as equivalent of ascorbic acid [55].

#### 3.4.4. Reducing Power

The reducing power assay consists of the reduction of the potassium ferricyanide by the samples. Briefly, the dextrans were mixed with a phosphate buffer 0.2 M (pH 6.6) and incubated with potassium ferricyanide (1% *m*/*v*) at 50 °C for 20 min. One solution of trichloroacetic acid (10% *m*/*v*) was used to stop the reaction. Distilled water and ferrous chloride (0.1% *m*/*v*) were added to the solution and the absorbing capacities were measured at 700 nm. Results were accounted as an activity percentage, considering the largest concentration of ascorbic acid (the standard) as 100% activity [43].

#### 3.4.5. Hydroxyl Radical Scavenging Activity Assay

The hydroxyl radical scavenging activity of the dextrans had its investigation based on the Fenton reaction. Hydroxyl radicals were generated by the reaction containing a sodium phosphate buffer 150 mM (pH 7.4) mixed with ferrous sulfate heptahydrate 10 mM, ethylenediaminetetraacetic acid (EDTA) 10 mM, sodium salicylate 2 mM and hydrogen peroxide 30%. Hydrogen peroxide was replaced with phosphate buffer for the blank sample. The solutions were incubated at 37 °C for 1 h and the scavenging capacity was detected via the absorbing capacity analysis at 510 nm. The results were recorded as scavenging percentage [24]. Gallic acid was used as positive control.

#### 3.4.6. Inhibition of Lipid Peroxidation

The assay investigated the oxidation of the β-carotene. Briefly, the dextrans were mixed with 0.5 mg of β-carotene and chloroform, 25 μL of linoleic acid and 200 mg of Tween 40. Initially, the chloroform was evaporated and 50 mL of distilled water saturated with O_2_ was added. The emulsions were incubated with the dextrans and the inhibition was detected by monitoring the absorbing capacities at 490 nm. The reactive mixture was incubated at 50 °C for 2 h and once again the absorbing capacities were verified. Butylated hydroxyanisole (BHA) was used as positive control. The results were recorded as inhibition percentage [56].

#### 3.4.7. Hydrogen Peroxide Scavenging Activity Assay (H_2_O_2_)

The assay was conducted to investigate the dextran’s capacity to scavenge the hydrogen peroxide. Briefly, the reaction was to mix the dextrans with 100 mL of H_2_O_2_ 0.002%. Then, 0.8 mL of sodium phosphate buffer 0.1 M and sodium chloride 100 mM were added. The solutions were incubated at 37 °C for 10 min. After that, 1 mL of the phenol red indicator (0.2 mg/mL) with 0.1 mg/mL of peroxidase in sodium phosphate buffer 0.1 M was added. After 15 min, 50 mL of sodium hydroxide 1 M were added. The absorbance was immediately measured at 610 nm, having the Gallic acid as positive control. Results were recorded as scavenging percentage [57]. All assays were performed three times in triplicate (*n* = 3).

#### 3.4.8. Superoxide Radical Scavenging Activity Assay

The superoxide radical scavenging activity of the dextrans was investigated through the inhibition of the photochemical reduction of the nitroblue tetrazolium (NBT) in the riboflavin-light-NBT system. The dextrans (50 mM) were added to a solution of phosphate buffer 50 mM (pH 7.8), riboflavin 2 mM, EDTA 100 mM, l-methionine 13 mM and NBT 75 mM. The formation of blue formazan was monitored by the increase in absorbance at 560 nm after the exposure to light for 10 min in a closed box. An identical reaction was maintained in the dark and served as a blank. Gallic acid was used as positive control. Results were recorded as scavenging percentage [43]. All assays were performed three times in triplicate (*n* = 3).

### 3.5. Mitochondrial Reduction of MTT

The cytotoxicity of the dextrans was evaluated following the mitochondrial reduction of the MTT. Briefly, the assay was conducted to investigate the cellular enzyme’s capacity of reducing the 3-(4,5-dimethylthiazol-2-yl)-2,5-diphenyltetrazolium bromide (MTT) to formazan in RAW 264.7 macrophages with an active metabolism. The cells were incubated with the dextrans (50 mM) at 37 °C in the presence of DMEM for 24 h. After incubation, traces of dextrans were removed by washing the cells twice with 200 μL PBS and applying MTT (1 mg/mL) dissolved in 100 μL of fresh medium to determine the effects of the samples on cell viability. Cells were then incubated for 4 h at 37 °C, in 5% CO_2_. The MTT-formazan product dissolved in 100 μL of ethanol was estimated by measuring the absorbance at 570 nm in a Multiskan Ascent Microplate Reader. Inhibition of MTT reduction is presented as a percentage of cell proliferation under no treatment conditions. Absorbance was measured at 570 nm [43]. 

### 3.6. Production of Nitric Oxide (NO)

To determine the effect of glucans in the amount of nitric oxide (NO) released by the RAW cells, the method described by Alves and collaborators [54] was used. Briefly, macrophages (RAW 264.7 cell line) were incubated (3 × 10^5^ cells/well) in culture plates of 24 wells at 37 °C in CO_2_ 5% both with and without lipopolysaccharides (LPS) in the presence of dextrans (50 mM). After 24 h a reaction with Griess reagent was conducted on the supernatant. The absorbance of the reaction was monitored at 540 nm. 

### 3.7. Anticoagulant Assays

The anticoagulant activity of the glucans was evaluated using two in vitro tests: the activated partial thromboplastin time (aPTT) and the prothrombin time (PT). Both assays were conducted in the 50 mM concentration for all samples. The aPTT and PT assays were conducted following specification of the manufacturers (Labtest, Sao Paulo, SP, Brazil) and each assay was performed three times in triplicate (*n* = 3).

#### 3.7.1. Activated Partial Thromboplastin Time (aPTT)

The coagulation assay via activated partial thromboplastin time (aPTT) was conducted using normal human plasma treated with citrate in which the dextrans were incubated. Briefly, dextrans were mixed with the plasma treated with citrate and then incubated at 37 °C. After 3 min, cephalin was added and the reaction was once again incubated. Following another 3 min, CaCl_2_ (100 μL, 20 mM) was added and the coagulation time was measured by the coagulometer. Clexane^®^ (Sanofi Aventis Farmacêutica Ltda, São Paulo, SP, Brazil) and normal human plasma treated with citrate were used both as standard and control, respectively [54]. All assays were performed three times in triplicate (*n* = 3).

#### 3.7.2. Prothrombin Time (PT)

The coagulation assay via prothrombin time (PT) was also conducted using normal human plasma treated with citrate, incubated along with the samples. Briefly, the dextrans were mixed with normal human plasma treated with citrate and incubated at 37 °C for 3 min. Then, soluplastin was added and the coagulation time was measured by the coagulometer [30].

### 3.8. Statistical Analyses

All data have been expressed as the average ± standard deviation. Statistical analyses were conducted via a simple variation analyses (one-way ANOVA) followed by the Tukey–Kramer (*p* < 0.05) test. All tests were conducted on the GraphPad Prism 5.01 (GraphPad Softwares, La Jolla, CA, USA).

## 4. Conclusions

Dextrans D10, D40 and D147 are the same type of polysaccharide and their only difference is their molecular weight. They are formed by glucose in the alpha configuration and are free of protein and phenolic contaminants. The same dextran molecules—but with different molecular weights—showed different antioxidant and immunomodulatory activities. In one antioxidant test, the dextran activity increases according to the increase of the molecular weight. In another test, however, the activity might decrease or show no correlation to the molecular weight. It can be further stated that antioxidant dextrans act mainly in the termination of the formation process for reactive species. The great highlight of this study was the D40 dextrans, which presented with antioxidant activity in most of the tests conducted and showed a greater inhibiting activity of the lipid peroxidation: it presented with a greater hydrogen peroxide scavenging capacity when compared to the two other dextrans and it was also the only one to present with immunomodulatory activity. This points to the observation that, in order to attain dextrans that present with excellent immunomodulatory and antioxidant activity, one must find dextrans with molecular weight around 40 kDa. However, more studies, tests and published data are required to confirm this hypothesis. Therefore, future studies hold the intent to best characterize the immunomodulatory and antioxidant activities of D40 dextrans—or other dextrans with similar molecular weights.

## Figures and Tables

**Table 1:** Chemical composition of the D10, D40 and D147 dextrans.

Dextrans	Total Sugar (%)	Proteins (%)	Phenolic Compounds (%)	Molar Ratio * (%)		
				Glc	Man	Gal
D10	91.3	n.d.	n.d.	1:0	0:0	0:0
D40	90.5	n.d.	n.d.	1:0	0:0	0:0
D147	93.1	n.d.	n.d.	1:0	0:0	0:0

Glc: glucose; Man: mannose; Gal: galactose. * Molar ratio obtained by high performance liquid chromatography (HPLC) analyses after acid hydrolysis (HCl 2 M; 2 h; 100 °C). n.d.: Not detectable in the evaluated conditions.
